# Reservoir Computing-Based Design of ZnO Memristor-Type Digital Identification Circuits

**DOI:** 10.3390/mi13101700

**Published:** 2022-10-10

**Authors:** Lixun Wang, Yuejun Zhang, Zhecheng Guo, Zhixin Wu, Xinhui Chen, Shimin Du

**Affiliations:** 1 Faculty of Electrical Engineering and Computer Science, Ningbo University, Ningbo 315211, China; 2 College of Information Engineering, Jinhua Polytechnic, Jinhua 321017, China; 3 College of Science & Technology, Ningbo University, Ningbo 315300, China

**Keywords:** Reservoir Computing, memristor, synaptic plasticity, stress activity

## Abstract

Reservoir Computing (RC) is a network architecture inspired by biological neural systems that maps time-dimensional input features to a high-dimensional space for computation. The key to hardware implementation of the RC system is whether sufficient reservoir states can be generated. In this paper, a laboratory-prepared zinc oxide (ZnO) memristor is reported and modeled. The device is found to have nonlinear dynamic responses and characteristics of simulating neurosynaptic long-term potentiation (LTP) and long-term depression (LTD). Based on this, a novel two-level RC structure based on the ZnO memristor is proposed. Novel synaptic encoding is used to maintain stress activity based on the characteristics of after-discharge and proneness to fatigue during synaptic transmission. This greatly alleviates the limitations of the self-attenuating characteristic reservoir of the duration and interval of the input signal. This makes the reservoir, in combination with a fully connected neural network, an ideal system for time series classification. The experimental results show that the recognition rate for the complete MNIST dataset is 95.08% when 35 neurons are present as hidden layers while achieving low training consumption.

## 1. Introduction

**Figure 1:**
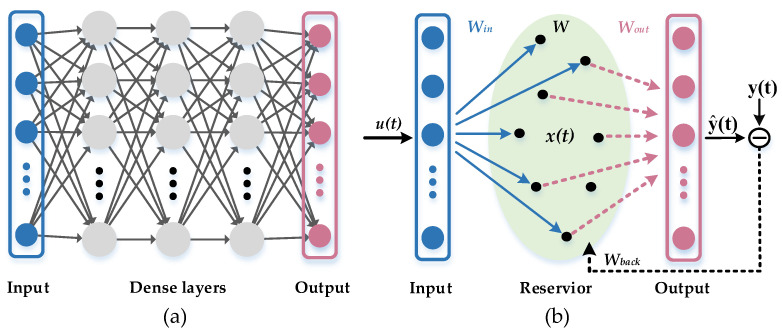
(**a**) Recursive neural network structure; (**b**) traditional echo state network structure.

As artificial neural networks (ANN) are widely used in the field of artificial intelligence, some of their limitations in the process of specific applications continue to emerge. For example, convolutional neural networks (CNNs), which consist of convolutional layers and fully connected layers [1], show strong recognition performance when processing static image information [2,3], but when the temporal relationship between each input vector is very tight, the recognition error of CNNs is greatly increased. Similar to general forward-structured neural networks, CNNs are not suitable to deal with problems related to time sequence. On the other hand, although recurrent neural networks (RNN) can be used to solve time series-related problems, they have disadvantages such as overly complex training algorithms, large computational effort, slow speed of convergence, and difficulty in determining the network structure, as shown in Figure 1a, in addition to the case of memory fading. These are serious obstacles to the application of recursive neural networks in practical problems.

To solve the problem of training consumption, Jaeger and Maass proposed echo state networks (ESNs) [4] and liquid state machines (LSMs) [5], respectively. Although they proposed different perspectives, their intention was to improve upon the traditional recurrent neural networks, and in 2007, they unified the name “Reservoir Computing” [6]. RC is a biologically inspired machine learning model that mainly simulates the neuronal activity patterns of the human brain excited by neural impulse signals during information processing. As shown in Figure 1b, the conventional RC structure mainly consists of an input layer, a reservoir layer, and an output layer. The input vector is connected to the reservoir via the input weight *W_in_*. The reservoir generally consists of a large number of randomly connected nonlinear nodes, with inter-node connection weights denoted by *W*. When the input changes, the RC behaves as a delayed response, and the output layer classifies the input signal by obtaining data from each nonlinear node and performing linear regression. Both *W_in_* and *W* are randomly generated throughout the system, and do not require further training once they are generated. Only the output weight *W_out_* needs to be trained before being obtained. Since the relationship between the state variables and the output is linear, the output weight *W_out_* only needs to be obtained by solving a linear regression problem, which is the biggest advantage of RC over the traditional RNN.

Presently, a large number of potential binary or ternary oxides have shown high-speed resistive switching states and large switching ratios [7,8,9], which is extremely beneficial for the development of applications of memristors [10]. When a scanning voltage or a pulsed voltage is applied to the memristor, the memristor exhibits continuous multi-conductor state changes, similar to the regulation of the connection strength of neural synapses, called synaptic plasticity. Several studies have already shown that this neuronal structure and transmission similar to those of the human brain have great advantages in building memristor neural networks, providing a promising non-von Neumann computational paradigm and, thus, a certain degree of significant speed and energy efficiency. In addition, memristors have made great progress not only in the hardware of neural networks [11,12,13,14,15,16,17], but also are widely used in many fields such as information storage [18,19], nonlinear dynamics [20,21], neuronal models [22,23], nonvolatile logic [24,25], and chaotic circuits [26,27,28]. Several research results have now shown the realizability of hardware RC systems, such as using spintronic oscillators [29,30], photonic techniques [31,32,33], or memristors [34,35,36,37,38]. Among them, the nonlinear dynamic response characteristics possessed by memristors are well-suited for RC system hardware [39,40], but this has certain limitations, such as the input signal duration and rate, which are strictly limited to a certain range based on the conductivity self-attenuation characteristic reservoir, which is far from satisfying the increasingly complex information-processing needs. In this paper, we demonstrate a novel RC structure based on ZnO memristors, utilizing the memristor conductance accumulation effect for initial processing of the input data and adjusting the reservoir state by controlling the mask. In addition, based on the nonlinear dynamic response of the memristor, pulse modulation is used to simulate the short-term memory effect of neurons and maintain their stress activity, thereby helping to greatly alleviate the limitations of the self-attenuating characteristic reservoir on the duration and interval of the input signal. Finally, the output of the memristor is directly used as the reservoir state, thus omitting the read pulse operation and improving the data-processing speed of the RC system. The experimental results show that the recognition rate for the complete MNIST dataset is 95.08% when 35 neurons are present as hidden layers.

## 2. Methods

In this paper, the ZnO memristor prepared in the laboratory was modeled, and a novel two-level RC structure was proposed based on this device. Meanwhile, due to the limited speed of information processing by the self-attenuating characteristic reservoir, the concept of after-discharge was introduced, and a novel encoding method was used to maintain the stress activity of simulated neurons to different pulses in order to improve the recognition accuracy.

### 2.1. Pt/ZnO/Pt Memristor and Model

**Figure 2:**
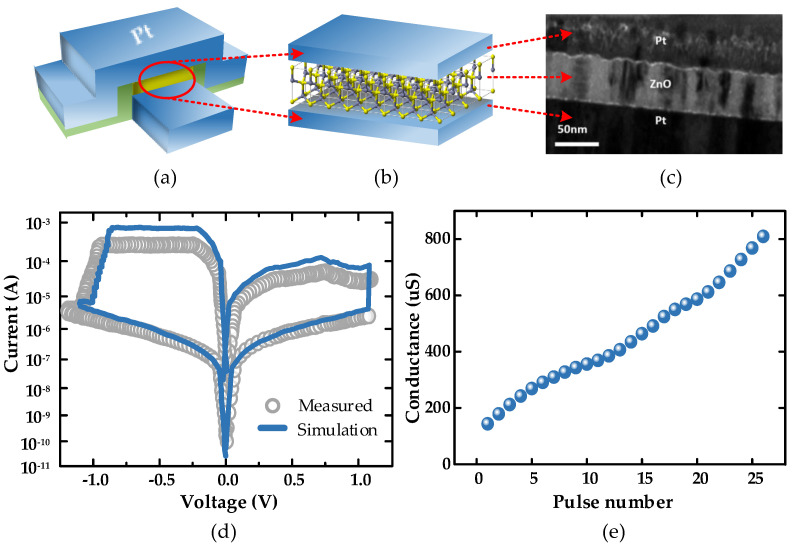
(**a**) Pt/ZnO/Pt memristor structure. A resistive switch consisting of a ZnO film is sandwiched between the top and bottom electrodes; (**b**) Pt/ZnO/Pt memristor showing a sandwich structure; (**c**) cross−sectional TEM image showing the Pt/ZnO/Pt memristor; (**d**) comparison of the experimental measurement data of the prepared memristor with the simulation data of the mathematical model; (**e**) Evolution of the device conductance as a function of the voltage pulse stressing numbers. All the voltage pulses show the same width of 200 ms and amplitude of 1.8 v.

Among all transition metal oxides, ZnO has the advantages of stable resistive switching characteristics, uniformity, low bias requirement for SET and RESET voltages, low processing cost, and low pollution, so ZnO was chosen as the resistive layer material for the memristor in this paper. Finally, a bipolar memristor with asymmetric resistive switching characteristics was formed [7,9]. Figure 2a,b shows the Pt/ZnO/Pt-structured memristor prepared in the laboratory, which first deposited a 50 nm thin film of ZnO as a resistive layer on a commercial Pt/Ti/SiO2 wafer using RF magnetron sputtering, and then deposited a 50 nm thick Pt electrode to form a Pt/ZnO/Pt structure using electron-beam deposition, where the specific fabrication process and device characteristics have been described in detail in a previous work [41]. Figure 2c shows the transmission electron microscopy image of the memristor cross section.

In general, memristors use the conductive mechanism of conductive filaments (CFs), which undergo a resistance state transition through ion migration and redox reactions. The device exhibited a distinct resistive switching behavior at a direct current (DC) scan voltage, as well as stable multi-state characteristics. A voltage was applied at the top electrode (TE), and the bottom electrode (TB) was grounded during the scan. When a positive bias was applied, the device changed from a high-resistance state (HRS) to a low-resistance state (LRS); we defined this resistance change of the device as STE; conversely, when a negative bias was applied, the device changed from a high-resistance state (LRS) to a low-resistance state (HRS); we defined this resistance change of the device as RESTE. After repeated experiments, the mathematical model was constructed based on the drift speed-adaptive memristor (DSAM) [42] based on the obtained data. The i–v relationship of DSAM model is shown in the following equation:

(1) $$v(t)=(R_{off}-x\Delta R)\cdot i(t)$$

where *R_on_* and *R_off_* are the minimum and maximum resistance values of the memristor, Δ*R* is their difference, and *x* is denoted as the normalized conducting zone width, the derivative of which is expressed as:

(2) $$\frac{dx}{dt}=g(i,v)\cdot f(x,i),$$

(3) $$g(i,v)=\left\{ \begin{array}{cc} k_{on}\cdot \Delta R\cdot i(t), & v(t)>v_{on};\\ 0, & v_{off}\leq v(t)\leq v_{on};\\ k_{on}\cdot \Delta R\cdot i(t), & v(t)<v_{off}, \end{array}\right.$$

(4) $$f(x,i)=\left\{ \begin{array}{cc} {\left(a\cdot (1-x)\right)}^{p}, & i>0;\\ {\left(a\cdot x\right)}^{p}, & i\leq 0. \end{array}\right.$$

where g(i,v) is the voltage threshold equation, f(x,i) is the velocity-adaptive equation, *v_on_* and *v_off_* denote positive and negative threshold voltages, *k_on_* and *k_off_* denote linear adjustable parameters, and *a* and *p* are fitting parameters.

**Figure 3:**
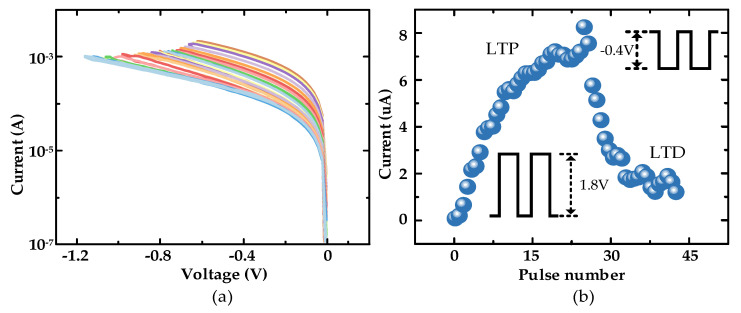
(**a**) Continuous regulation of the device current in the negatively biased reset processes; (**b**) Simulation of bionic synaptic LTP and LTD characteristics (pulse width 200 ms and pulse interval 50 ms).

According to the data obtained for fitting, the main parameters of the final model were set to *v_on_* = 0.2 v, *v_off_* = −0.2 v, *R_on_* = 250 Ω, *R_off_* = 10 k, *k_on_* = 270, *k_off_* = 24, *a* = 0.47, and *p* = 2.3. As shown in Figure 2d, the proposed mathematical model of the memristor had a high agreement with the actual device in terms of current–voltage relationship under the same programming pulse. As shown in Figure 2e, when a positive pulse with a voltage of 1.8 v was applied, the memristor exhibited multiple continuously adjustable stable conductance states. However, since positive feedback during SET usually leads to uncontrolled excessive growth of CFs, if a negative pulse with an amplitude of 1.8 v is applied during the execution of RESET, this will result in the inability to achieve the transition between stable conductance states. Therefore, during RESET, it was necessary to change the cutoff voltage and adjust the CFs in a relatively mild way to achieve the transition between resistive states during RESET. According to the reset operation of the memristor in DC scan mode shown in Figure 3a, it was found that the device currents were all decreasing, so a relatively mild 0.4 v was used as the reset voltage.

On the other hand, according to the above device’s performance, shown in Figure 3b, its resistance state with a continuous adjustable characteristic was highly similar to a biological neural synapse. According to the Hebbian learning rule, when stimulation of the presynaptic membrane precedes the postsynaptic membrane, the postsynaptic current tends to rise and the synapse shows LTP; conversely, the postsynaptic current tends to fall and the synapse shows LTD [43]. Accordingly, the pulses were applied to the top and bottom electrodes of the memristor, respectively, causing different degrees of resistance state changes in the memristor, and based on this property, the following RC system was constructed by simulating the bionic synapse.

### 2.2. RC System Based on ZnO Memristor

The echo state network structure consists of sparsely connected large-scale analog neurons, and its structure is shown in Figure 1b. The network structure consists of *M* inputs, *N* nodes, and *P* outputs; the input vector, state vector, and output vector after sampling are expressed as follows:

(5) $$\left\{ \begin{array}{c} u(n)={\left[u_{1}\left(n\right),\;u_{2}\left(n\right),\;\ldots ,\;u_{M}\left(n\right)\right]}^{T}\\ x(n)={\left[x_{1}\left(n\right),\;x_{2}\left(n\right),\;\ldots ,\;x_{N}\left(n\right)\right]}^{T}\\ y(n)={\left[y_{1}\left(n\right),\;y_{2}\left(n\right),\;\ldots ,\;y_{P}\left(n\right)\right]}^{T} \end{array}\right.$$

In this structure, ESNs map u(t) to a higher-dimensional space by input weights, while to eliminate the influence of arbitrary initial states on the dynamic characteristics of the system, the system states are collected only after a certain moment *m*, which is finally classified by the output layer for data classification.

The output weights *W_out_* are trained by computing the error between the network output value $\hat{y}(t)$ and the desired output y(t) [44,45], with the aim of minimizing the error.

(6) $$\hat{y}(t)=\sum_{i=0}^{P-1}x_{i}(n)W_{i}^{out}(n)$$

(7) $$E=\min\frac{1}{Z-z+1}{\sum_{j=z}^{Z}\left[\sum_{i=0}^{P-1}x_{i}(n)W_{i}^{out}(n)-y_{i}(n)\right]}^{2}$$

where *Z* − *z* + 1 is the length of the sampled data. The state equation and the output equation during training [46] can be expressed as:

(8) $$\left\{ \begin{array}{cc} x\left(t+1\right) & =f\left[u\left(t\right)W_{in}+x\left(t\right)W+f_{back}\left[\hat{y}\left(t\right)-y\left(t\right)\right]W_{out}\right]\\ \hat{y}\left(t+1\right) & =u\left(t\right)W_{in}+x\left(t+1\right)W+f_{back}\left[\hat{y}\left(t\right)-y\left(t\right)\right]W_{out} \end{array}\right.$$

The above equation ultimately requires solving for the connection weights *W_out_* between the reservoir and the output, while the outputs y(t) are generated by the state variables x(t) through a fully connected neural network, which exhibits a linear relationship. At the mathematical level, it is simply necessary to solve the linear regression problem.

**Figure 4:**
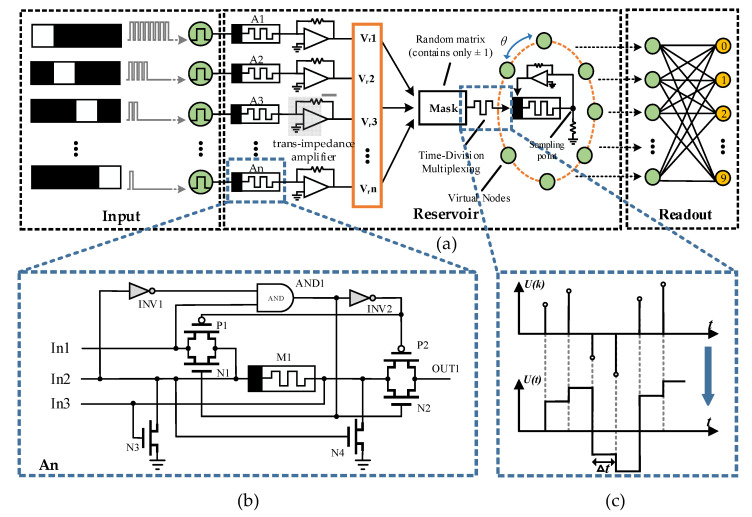
(**a**) RC system structure proposed in this paper; (**b**) Part of the peripheral circuit of the memristor in the first-level reservoir; (**c**) Hold operation is performed on each voltage signal to keep the voltage stable in the Δt range.

Based on the above derivation, the structure diagram of the RC system designed in this paper is shown in Figure 4a. To recognize pictures using the RC system, the first step was to turn the pictures into a circuit-recognizable form, i.e., to encode the pixels. The image input to the reservoir was cropped to an *n* × 4 format, where each line contains 4 pixels, with black pixels representing the number “0” and white pixels representing the number “1”, resulting in a 4-bit binary number. When the highest bit was high (white pixel), the pixel was encoded as 8 voltage pulses; when the lowest bit was high, the pixel was encoded as 1 voltage pulse, and so on, for a total of 16 arrangements.

From Figure 2e, it can be seen that the memristor conductance state was positively correlated with the number of input pulses, and different numbers of pulses led to different conductance states. Specifically, when a positive pulse was applied to the memristor, the memristor state changed and the response showed an increase in conductance. When multiple pulses were applied at short intervals, the conductance kept increasing, while the memristor state returned to the original state when multiple reverse voltages were applied continuously, i.e., the resistance state when no pulses were applied. Figure 4b shows part of the peripheral circuitry of the memristor in the first level of the reservoir. When the pulse sequences corresponding to the different pixel arrangements are input from the IN2 port, the two transmission gates close, N3 closes, N4 conducts, and the voltage pulses pass through the memristor and are grounded. At this time, the image information corresponding to the pulse sequence is stored in the memristor, and a read pulse is output from IN1 to read the memristor resistance value. When IN1 is high, the two transmission gates are turned on by the inverter and with the AND gate, and both N3 and N4 are turned off, so the output current is output from OUT1 after the read pulse passes through the memristor. After a group of data is processed, the memristor resistance needs to be reset; at this time, the reset pulse is output by IN3, the same two transmission gates are closed, N3 is on and N4 is off, and the pulse will reset the memristor resistance and then ground through N3. This prevents any current from passing through the transmission gate and affecting the RC system. In this pixel-encoding approach, the original set of image features is then represented as the conductance state of individual memristors after pulse stimulation, and the collective memristor state contains all pixel point information. Since the ZnO memristor is a voltage-controlled device, the current signal is converted into a voltage form using the transimpedance amplifier to facilitate subsequent processing. Subsequently, all voltage signals are integrated into a vector and multiplied with a matrix containing only ±1 (for reasons described in the next section). Finally, the voltage signal is stabilized within Δt (Δt is the transfer delay of the memristor) by performing the operation shown in Figure 4c, generating a time pulse sequence.

The traditional reservoir is composed of randomly connected nonlinear neuron nodes. The concept of “after-discharge” in synaptic transmission is introduced here, so that the device state depends not only on the current input, but also on the availability of other pulse inputs within the nearest τ time frame through a feedback circuit. Due to the transfer delay, the interaction between neurons prevented the excitation response from disappearing immediately in the reservoir, thus forming rich reservoir states at each nonlinear node. However, the hardware implementation of the reservoir process is very difficult to achieve random connections between multiple devices and guarantee their performance, so this paper used the time-division multiplexing principle to input the pulse sequence into the series circuit of the memristor and resistor. The pulse sequence, as shown in Figure 4c, will result in the memristor having different conductance states at different time periods, so the time required for the memristor to go from LTP to LTD under the same pulse conditions was defined as τ. We divided τ into *N* equidistant points (node spacing θ=τ/n) corresponding to *N* virtual nodes, and the bottom electrode of the memristor was used as the sampling point on the virtual node for sampling. It should be noted that there was no need to distinguish between the voltage signal of the input and the output signal of the feedback circuit separately, but there was a need to directly collect the state changes of the memristor at different points in time under the combined effect of these two signals, where virtual nodes were used instead of the nonlinear nodes in the traditional reservoir, and the collected data could be linearly regressed to characterize the original corresponding image information.

The last point to note is that the delay error existing in the whole reservoir structure mainly came from two aspects, namely the circuit delay and sampling delay. Firstly, the delay existing after the hardware circuit was completed was relatively fixed, and the impact on the reservoir was also relatively fixed, so it had little impact on the final recognition accuracy; secondly, in terms of sampling, since the sampling was done by external mature commercial ADC modules, the sampling delay was relatively small. Therefore, the biggest time error came from the fact that the time point at which each sampling started could not be precisely synchronized, but the state change of the same image information was convergent after it was inputted into the reservoir; the different starting time of sampling only led to a deviation in the time dimension of the collected data, but the actual information content was the same, so the impact on the final recognition accuracy was also small.

### 2.3. Synaptic Plasticity-Based Coding

**Figure 5:**
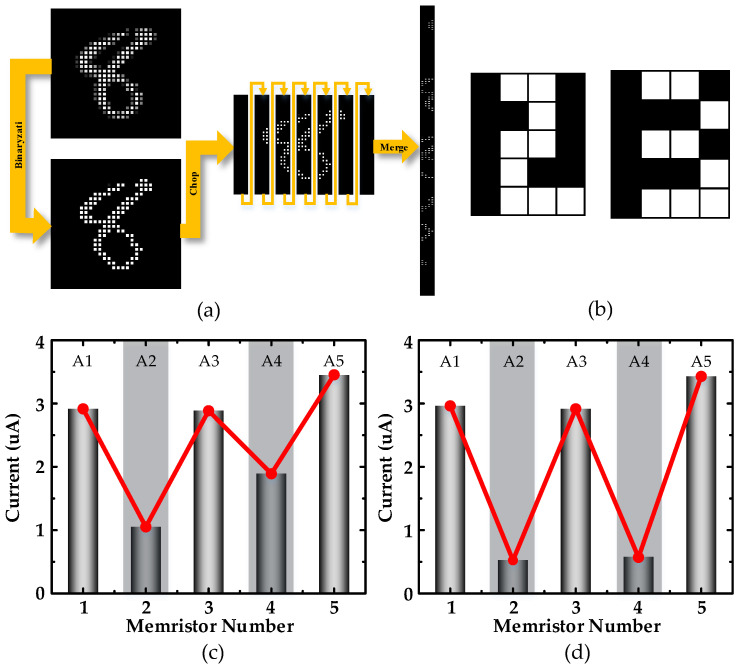
(**a**) Grayscale image conversion to binary image with 7 columns sliced and stitched together. In the end, the image is transformed into a 196 × 4 image and encoded; (**b**) example images of numbers “2” and “3”; (**c**) reservoir state of “2”; (**d**) reservoir state of “3”. A1-5 corresponds to A1-n in Figure 4a (at this point n = 5).

In this section, the picture pre-processing method and the pulse adjustment method for the memristor are explained separately. Because the advantage of the RC lies in the processing of temporal information, in this paper, the MNIST dataset was transformed into temporal information before processing and recognition, as shown in Figure 5a, requiring pre-processing of 28 × 28 images. Firstly, the original grayscale image was converted into a binary image. At this point, if a whole row was treated as a time series, there were, theoretically, 1,048,576 different inputs, which are difficult to distinguish for a single memristor, so in order to improve the recognition accuracy, the image was cut into 7 columns (each column was 28 × 4 in size) on average, and subsequently integrated into a 196×4 image. According to the encoding method described in the previous section, the images were converted into pulse sequence classes, and the differences existing in the original images were distinguished using the different resistance states of the devices due to different number of pulses. Based on this feature, a simple example is shown in Figure 5b, where the image size is 5 × 4 and the contents are the numbers “2” and “3”. Here, five memristors are used to recognize the images, and each row in both images corresponds to the same number of white pixels, with rows 1 through 5 being 2, 1, 2, 1, and 3, respectively. The pixel information is converted into temporal pulses that are input into the memristors, whose reservoir states are characterized in Figure 5c,d, respectively. There was a significant difference in the state of the reservoir when only the pixel arrangement was different; thus, it was shown that the memristor had a natural advantage for recognizing time-sensitive sequences.

**Figure 6:**
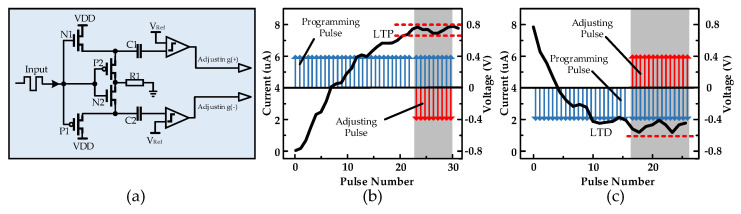
(**a**) Schematics for generating auto−regulating pulses; (**b**) Continuous positive pulses are input to adjust the memristor to the LTP state. If the subsequent programmed pulses remain positive, a reverse regulation pulse is automatically applied to keep the resistance state stable; (**c**) Continuous negative pulses are input to adjust the memristor to the LTD state. If the subsequent programmed pulses remain negative, a reverse regulation pulse is automatically applied to keep the resistance state stable.

Although the above process could perform preliminary classification of the temporal pulse signal, in this coding method, the inputs were positive pulses, and the memristor resistance range was limited; when the memristor was adjusted to the LTP state, it could no longer respond to the pulse stimulus, so the data collected at the sampling point did not reflect the input difference, and eventually led to a network recognition accuracy with substantial error, so using this method alone does not apply to complex image recognition. The key to improving accuracy is to maintain synaptic stress activity in response to pulses, so this paper multiplied the voltage signal in a second-stage reservoir with a matrix containing only ±1 to assign random positive and negative signs to the pulse sequence, minimizing the device being regulated all the way to LTP or LTD. At the same time, a synaptic plasticity-based encoding approach was introduced to maintain synaptic stress activity in response to impulses. The circuit diagram for generating the regulation pulses is shown in Figure 6a. This circuit works simultaneously and independently of the sampling circuit described above, and the input signal shares the signal shown in Figure 4c. When continuous positive pulses are input, N1 and N2 turn on, P1 and P2 turn off, then VDD charges C1 through the N1 transistor, and when the number of positive pulses reaches a certain number, the voltage across C1 capacitor exceeds the reference voltage Vref, then the comparator outputs a high-level pulse and connects to the bottom electrode of the memristor for the purpose of regulating the conductance; conversely, the regulating pulse is output from another comparator and connects to the top electrode of the memristor. If a low-level pulse is input at this time, N1 and N2 turn off, P1 and P2 turn on, and VDD charges C2 while discharging C1 through P2. However, the amount of conductance regulated by a large number of forward pulses cannot be offset by one reverse pulse and, similarly, the amount of charge accumulated by a large number of forward pulses cannot be fully released by one reverse pulse, so a large resistor R1 needs to be connected in series during the discharge process for slowing down the discharge of the capacitor.

With continuous input of forwarding programming pulses (blue arrows), as shown in Figure 6b, the memristor conductance continuously increases, manifesting as an LTP state, and if the input remains a positive pulse, reverse regulation pulses (red arrows) are punched in after each time frame to stabilize the conductance within a certain range. This is manifested by the fact that when a continuous positive pulse is input, the conductance of the memristor is regulated to a certain value and then no longer increases and no longer responds routinely to similar pulses, which is biologically manifested as a fatigue state due to the depletion of transmitters in the synaptic transmission process. Similarly, as shown in Figure 6c, when the memristor behaves as an LTD state, with the input remaining as negative pulses, positive pulses are punched in after each time frame to prevent the conductance from continuing to decrease, no longer responding to the same kind of negative pulses, but at the same time maintaining a high sensitivity to non-identical pulses to characterize the input difference. By adopting this new encoding method to maintain the stress activity of simulated synapses to pulses, this will cause different changes in the resistance state of the amnestic at different moments when pulses containing image information are input into the memristor, and the collected data will reflect the amount of pulse changes more obviously, ultimately improving the recognition accuracy.

## 3. Results

Based on the above method, the MNIST dataset is recognized using a ZnO memristor-based RC system. The MNIST dataset in the network simulation is obtained from the National Institute of Standards and Technology (NIST) database, where 60,000 images are used for training and 10,000 images are used to test the network recognition accuracy. Because the advantage of the reservoir calculation lies in the processing of the time series information, in order to characterize the reservoir calculation, the MNIST dataset is transformed into time series information before processing and identification in this paper. 

**Figure 7:**
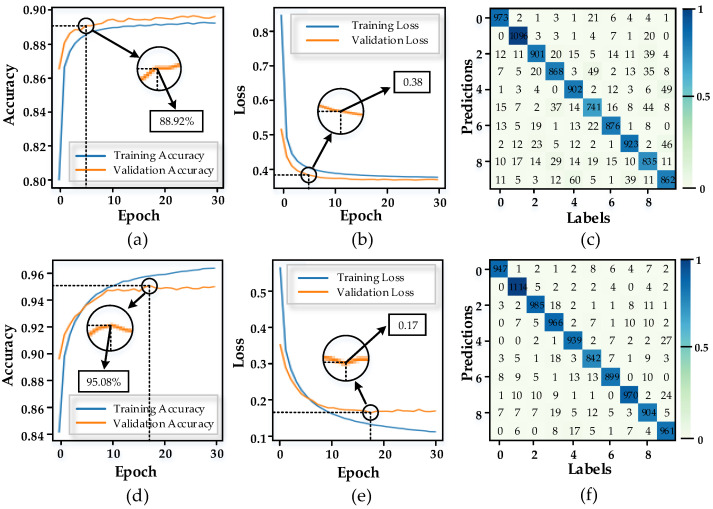
(**a**) Accuracy of training and validation (without hidden layers); (**b**) loss of training and validation (without hidden layers); (**c**) confusion matrix of data sets in (**a**); (**d**) accuracy of training and validation (hidden layer contains 35 neurons); (**e**) loss of training and validation (hidden layer contains 35 neurons); (**f**) confusion matrix of data sets in (**d**).

As shown in Figure 7a,b, even when the hidden layer is not included, the recognition accuracy of the complete MNIST dataset improved rapidly, reaching 88.92% after only five batches, with the loss function reduced to less than 0.4 (the loss function indicates the difference between the predicted and actual values, which is represented here by the mean squared error). In Figure 7c, the results of the MNIST test set are shown. The confusion matrix shows that the network has a high recognition accuracy for the dataset, with the main error being the recognition of “9” as “4”, followed by “5“ being incorrectly recognized as “8”. Furthermore, the network recognition is improved when 35 neurons are added to the readout layer as a hidden layer, and in Figure 7d,e, the network recognition accuracy reaches 95.08%, while the loss function is reduced to 0.17. The confusion matrix in Figure 7f shows that most of the errors are found in the recognition of “9” as “4”. Finally, since the results of the weights are not unique for each training, the two output layers are tested 20 times in this paper, and the final standard deviations regarding the recognition accuracy are 0.067 and 0.208, respectively.

Based on the above experimental results, the recognition performance of different RC systems for MNIST datasets is statistically presented in Table 1. First, in comparison with the traditional basic RC system [47], the recognition accuracy of the present design reaches 92% with the same dataset, but with simultaneous training parameters up to 528,000. Longitudinal comparison of training parameters and accuracy rates clearly shows that the hardware-implemented RC system has certain superiority. Secondly, in comparison with the dynamic memristor-based RC system [34], although the trainable parameters are lower than the present design, it uses an incomplete dataset with only 2000 images in the test set. The experimental results after selection are somewhat coincidental, and the cropping of images also simplifies the recognition difficulty to some extent. Nevertheless, the RC system proposed in this paper is still nearly 4% higher in recognition accuracy. Finally, in comparison with the diffusive memristor-based RC system [36], the recognition accuracy of the present design is also improved by 6.52% with superior image size and trainable parameters. In addition, the reservoir structure proposed in this paper has good performance when compared with other types of RC systems. Firstly, regarding the RC system based on photonic memristors [48], although photonic technology can achieve relatively low energy consumption, using this technology is still in its infancy and the processing power of the RC system is still far from being comparable to that of neural networks. Secondly, regarding the RC system based on the temporal kernel of the memristor [49], the performance of this reservoir is similar to the present work, but the RC system proposed in this paper has a slight advantage when the hidden layer is also added to the output network. The last is an RC system using a self-organizing nanowire network structure [50], and the final recognition accuracy of the method is 90.04%. Overall, the novel two-level RC structure based on ZnO memristors maintains high recognition accuracy for the complete MNIST dataset while achieving low training consumption.

## 4. Discussion

In this paper, a Pt/ZnO/Pt-structured memristor is used to implement a two-level RC system. First, the pulse sequences are classified by multiple parallel memristors, where multiple parallel memristors are seen as the first level of the reservoir. Subsequently, the individual memristor outputs are integrated into a vector and multiplied with a random matrix. Finally, the signal is fed into a series circuit of memristors and resistors through a simple time-division multiplexing process, i.e., the second-level reservoir. Through experiments and simulations, it is demonstrated that the use of simulated synaptic LTP and LTD properties in the second-level reservoir, combined with the after-discharge and easy-fatigue characteristics of the synaptic transmission process, greatly alleviates the input signal duration and interval limitations of the reservoir with self-decaying characteristics. Subsequently, by choosing the appropriate sampling frequency and power supply range, the timing signal can be processed effectively with an identification accuracy of 95.08% for the complete MNIST dataset. Theoretically, the system has a very large optimization space for the handwritten digit recognition task, which leads to error classification mainly due to the loss of original information in the process of grayscale image binarization, with varying degrees of influence of the pulse width and amplitude settings programmed by the device on the feature extraction. Meanwhile, in the error analysis, it is found that the probability of occurrence of different pixel arrangements is not the same, with the probability of occurrence of “0000” being as high as 77.2017% and that of “1111” being 4.5482%. The eight pulses with the highest probability of occurrence account for 50% of the total pulse types, but their share in the total number of occurrences is already as high as 98.4374%. Therefore, the subsequent optimization of the system focuses on the high-frequency pulses without spending a lot of resources to accurately classify all 16 types of pulses, thus effectively improving the recognition accuracy under constrained resource consumption.

Since RC encodes spatial information in the space–time domain without requiring training for physical reservoirs, this CMOS process-compatible architecture reduces network size and training costs, and is also attractive for applications that do not require extremely high processing speed but have strong constraints on memory size and computational power. These results provide a possible new idea for the physical implementation of brain-like computing.

## Figures and Tables

**Table 1:** Comparison of the recognition performance of RC system on MNIST dataset.

RC System Types	Number of Samples	Image Size	Trainable Parameters	Accuracy
This work	60,000/10,000	28 × 28	1970	89.52%
			7225	95.08%
Basic RC	60,000/10,000	28 × 28	528,000	92.0%
Dynamic memristor-based RC	14,000/2000	22 × 20	1760	85.6%
Diffusive memristor-based RC	60,000/10,000	22 × 20	2200	83.0%
Photonic quantum memristor-based RC	1000/1000 (Contains only 0,3,8)	18 × 12	about 1600	95%
Memristor temporal kernel-based RC	50,000/10,000	28 × 28	1970	90.01%
			7828	95.01%
Self-organizing nanowire network- based RC	60,000/10,000	28 × 28	-	90.04%

## Data Availability

Not applicable.
